# Metropolitan Hospital Sunday Fund, 1891

**Published:** 1891-08-01

**Authors:** 


### Metropolitan Hospital Sunday Fund, 1891.

The following is the Report of the Committee of Distribution adopted by the Council on Monday, 27th ult., the Right Hon. James Savory, Lord Mayor, in the chair :—

Your Committee have the honour to recommend the payment of awards to the 176 institutions enumerated below, being seven more than last year, and an increase of 71 since the first awards were made in 1873. The total amount available for distribution, after allowing for liabilities and the usual current expenses, is £43,157 15s. 4d. Of this total £40,907 15s. 4d. is now recommended to 120 hospitals and 56 dispensaries. Five per cent. of the total collected—viz., £2,250—is set apart to purchase surgical appliances. Your Committee recommend that all payments to the Fund after this date be carried to the credit of next year's Fund.

In compliance with an order of the Council, and for the special use of its members, tables of statistics have been prepared as usual, showing an analysis of the number of beds in hospitals, the cost of patients both at hospitals and dispensaries, the proportionate expense of management, as well as other valuable information, and copies are sent to every member of the Council.

The number of deputations representing committees of various hospitals, &c., invited to confer with your Committee, and to offer explanations on matters of apparently unsatisfactory character, was nineteen. Of these, nine elected to leave their awards entirely in the hands of this Committee. Ten were invited to send deputations to meet your Committee: nine attended, and of these four are recommended for awards on their full bases, five on a reduced scale, and one was not considered to be entitled to anything; including this hospital no awards are made to four applicants. One of the last-mentioned four could not receive an award under the laws of the Constitution.

The Bath Mineral Water Hospital is this year awarded in so far as it has provided for London patients. Other institutions which, being neither hospitals nor dispensaries, are rejected as being ineligible. One hospital, one dispensary, and two convalescent hospitals sent in their statistics too late to be considered.

### PARTICULARS OF THE AWARDS FOR 1891.

### Twenty-two General Hospitals.

Charing Cross Hospital, £1,083 6s. 8d.; French Hospital, £227 10s.; German Hospital, £704 3s. 4d.; Great Northern Central Hospital, £325; Guy's Hospital, £541 13s. 4d.; Italian Hospital, £97 10s.; King's College Hospital, £1,462 10s.; London Hospital, £3,250; Metropolitan Hospital, £433 6s. 8d.; Miller Hospital and Royal Kent Dispensary, £303 6s. 8d.; North-West London Hospital, £325; Poplar Hospital, £227 10s.; Royal Free Hospital, £975; St. George's Hospital, £1,625; SS. John and Elizabeth Hospital, £130; St. Mary's Hospital, £2,[illegible]20 16s. 8d.; Seamen's Hospital Society, £758 6s. 8d.; The Middlesex Hospital, £2,058 6s. 8d.; Training Hospital, Tottenham, £325; University College Hospital, £1,310 16s. 8d.; West London Hospital, £606 13s. 4d.; Westminster Hospital, £1,083 6s. 8d.

### Special Hospitals.

Five Chest Hospitals.—City of London Hospital for Diseases of the Chest, Victoria Park, £975; Hospital for Consumption, Brompton, £1,516 13s. 4d.; North London Consumption Hospital, Hampstead, £390; Royal Hospital for Diseases of the Chest, City Road, £379 3s. 4d.; Royal National Hospital for Consumption (Ventnor), £292 10s.

Twelve Children's Hospitals.—Alexander Hospital for Hip Disease in Childhood, W.C., £216 13s. 4d.; Belgrave Hospital for Children, S.W., £151 13s. 4d.; Cheyne Hospital for Incurable Children, S.W., £140 16s. 8d.; East London Hospital for Children, Shadwell, E., £574 3s. 4d.; Evelina Hospital for Sick Children, Southwark, S.E., £520; Home for Incurable Children, Maida Vale, W., £81 5s.; Home for Sick Children, Sydenham, S.E., £151 13s. 4d.; Hospital for Sick Children, Great Ormond Street, W.C., £823 6s. 8d.; North-Eastern Hospital for Children, Hackney Road, N.E., £368 6s. 8d.; Paddington Green Hospital for Children, W., £173 6s. 8d.; Victoria Hospital for Children, King's Road, Chelsea, S.W., £411 13s. 4d.; "The Vine," Sevenoaks, £37 18s. 4d.

Four Lying-in Hospitals.—British Lying-in Hospital, Endell Street, W.C., £48 15s.; City of London Lying-in Hospital, City Road, E.C., £75 16s. 8d.; General Lying-in Hospital, Lambeth, S.E., £75 16s. 8d.; Queen Charlotte's Lying-in Hospital, Marylebone Road, W., £325.

Six Hospitals for Women.—Chelsea Hospital for Women, S.W., £216 13s. 4d.; Hospital for Women, Soho Square, W., £455; Grosvenor Hospital for Women and Children, Vincent Square, S.W., £65; New Hospital for Women, Euston Road, W.C., £140 16s. 8d.; Royal Hospital for Children and Women, Lambeth, S.E., £216 13s. 4d.; Samaritan Free Hospital, Lower Seymour Street, W., £514 11s. 8d.

### Twenty-nine other Special Hospitals.

Cancer Hospital, Brompton, S.W., £541 13s. 4d.; London Fever Hospital, Islington, N., £433 6s. 8d.; Gordon Hospital for Fistula, Vauxhall Bridge Road, S.W., £43 6s 8d; St. Mark's Hospital for Fistula, City Road, E.C., £119 3s. 4d.; National Hospital for the Diseases of the Heart and Paralysis, Soho Square W., £108 6s. 8d.; Female Lock Hospital, Harrow Road, W., £297 18s. 4d.; Male Lock Hospital, Soho, W., £48 15s.; Hospital for Epilepsy, Paralysis, and other Diseases of the Nervous System, Regent's Park, N.W., £86 13s. 4d.; National Hospital for the Paralyzed and Epileptic, W.C., £682 10s.; West End Hospital for Diseases of the Nervous System, W., nil; Central London Ophthalmic Hospital, Gray's Inn Road, W.C., £54 3s. 4d.; Royal London Ophthalmic Hospital, Moorfields, E.C., £595 16s. 8d.; Royal South London Ophthalmic Hospital, St. George's Circus, S.E., £81 5s.; Royal Westminster Ophthalmic Hospital, Charing Cross, W.C., £86 13s. 4d.; Western Ophthalmic Hospital Marylebone Road, W., £15 3s. 8d.; City Orthopædic Hospital, Hatton Garden, E.C., £65; National Orthopædic Hospital, Great Portland Street, W., £43 6s. 8d.; Royal Orthopædic Hospital, Oxford Street, W., £97 10s.; Hospital for Diseases of the Skin, Stamford Street, S.E., £21 13s. 4d.; London Skin Hospital, Cranbourn-street, nil; St. Peter's Hospital for Stone, Covent Garden, £21 13s. 4d.; Central London Throat and Ear Hospital, Gray's Inn Road, W.C., £54 3s. 4d.; Hospital for Diseases of the Throat, Golden Squure, W.C., nil.; London Throat Hospital, Great Portland Street, nil.; Royal Ear Hospital, Frith Street W., £4 6s. 8d.; London Homœopathic Hospital, Great Ormond Street, W.C., £157 1s. 8d.; London Temperance Hospital, Hampstead Road, N.W., £498 6s. 8d.; Dental Hospital of London, Leicester Square, W.C., £113 15s.; National Dental Hospital, 149, Great Portland Street, W., 16s. 5d.

### Twenty-one Convalescent Hospitals.

Metropolitan Convalescent Institution, Sackville Street, W., £704 3s. 4d.; Bexhill Convalescent Home, Sackville Street, W., £325; All Saints' Convalescent Hospital, Eastbourne, £585; Mrs. Gladstone's Convalescent Home, Woodford, £140 16s. 8d.; Hanwell Convalescent Home, £21 13s. 4d.; Herbert Convalescent Home, Bournemouth, £43 6s. 8d.; King's College Hospital Convalescent Home, Hemel Hempstead, £108 6s. 8d.; Mrs. Kitto's Convalescent Home, Reigate, £86 13s. 4d.; Mrs. Marshman's London and Brighton Female Convalescent Home, £75 16s. 8d.; Mary Wardell Convalescent Home for Scarlet Fever, £140 16s. 8d.; Morley House Convalescent Home for Working Men, £54 3s. 4d.; Princess Frederica's Convalescent Home, East Molesey, £43 6s. 8d.; Children's Convalescent Home, Ramsgate, £21 13s. 4d.; St. Andrew's Convalescent Hospital, Clewer, £173 6s. 8d.; St. Andrew's Convalescent Home, Folkestone, £184 3s. 4d.; St. Barnabas Convalescent Home, Ramsgate, £21 13s. 4d.; St. John's Home for Convalescent and Crippled Children, Brighton, £21 13s. 4d.; Convalescent Home for Poor Children, St. Leonards-on Sea, £108 6s. 8d.; St. Mary Magdalene's Convalescent Home, Paddington, £54 3s. 4d.; St. Michael's Convalescent Home, Westgate-on-Sea, £48 15s., Seaside Convalescent Hospital, Seaford, £200 8s. 4d.

### Twelve Cottage Hospitals.

Beckenham Cottage Hospital, £54 3s. 4d.; Blackheath and Charlton Cottage Hospital, £65; Burstead Cottage Convalescent Home for Children, £21 13s. 4d.; Chislehurst, Sidcup, and Cray Valley Cottage Hospital, £54 3s. 4d.; Eltham Cottage Hospital, £29 5s.; Enfield Cottage Hospital, £37 18s. 4d.; Epsom and Ewell Cottage Hospital, £54 3s. 4d.; Hatfield Broad-Oak Cottage Hospital, £5 8s. 4d.; Reigate and Redhill Cottage Hospital, £70 8s. 4d.; Shedfield Cottage Hospital and Convalescent Home, £5 8s. 4d.; Sidcup Cottage Hospital, £32 10s.; Wimbledon Cottage Hospital, £43 6s. 8d.

### Nine Institutions.

Establishment for Gentlewomen, Harley Street, £86. 13s. 4d.; Hampstead Home Hospital and Nursing Institute, £92 1s. 8d.; National Sanatorium for Consumption, Bournemouth, £75 16s. 8d.; Royal Sea Bathing Infirmary, Margate, £628 6s. 8d.; Invalid Asylum, Stoke Newington, £81 5s.; Firs Home, Bournemouth, £32 10s.; St. Catherine's Home, Ventnor, £54 3s. 4d.; Hahnemann's Convalescent Home, Bournemouth, £21 13s. 4d.; Royal Mineral-Water Hospital, Bath, £37 18s. 4d.

### Fifty-Six Dispensaries.

Battersea Provident Dispensary, £65; Brixton, &c., Dispensary, £48 15s.; Brompton Provident Dispensary, £27 1s. 8d.; Camberwell Provident Dispensary, £102 18s. 4d.; Camden Provident Dispensary, £11 18s. 4d.; Chelsea, Brompton, and Belgrave Dispensary, £43 6s. 8d.; Child's Hill Provident Dispensary, £10 16s. 8d.; City Dispensary, £108 6s. 8d.; City of London and East London Dispensary, £27 1s. 8d.; Clapham General and Provident Dispensary, £37 18s. 4d.; Eastern Dispensary, £32 10s; Farringdon General Dispensary, £45 10s.; Finsbury Dispensary, £62 16s. 8d.; Forest Hill Dispensary, £37 18s. 4d.; Gipsy Hill and Upper Norwood Dispensary, £16 5s.; Hackney Provident Dispensary, £6 10s.; Hampstead Provident Dispensary, £54 3s. 4d.; Holloway and North Islington Dispensary, £81 5s.; Islington Dispensary, £54 3s. 4d.; Kensal Town Provident Dispensary, £14 1s. 8d.; Kensington Dispensary, £75 16s. 8d.; Maida Vale and St. John's Wood Dispensary, Kilburn, £48 15s.; Kilburn Provident Medical Institution, £32 10s.; London Dispensary, £21 13s. 4d.; London Medical Mission, Endell Street, £65; Margaret Street Infirmary for Consumption, £13; Marylebone Medical Mission and Dispensary, £37 18s. 4d.; Metropolitan Dispensary, £37 18s. 4d.; Notting Hill Dispensary and Maternity (Provident), £27 1s. 8d.; Paddington Provident Dispensary, £43 6s. 8d.; Pimlico Provident Dispensary (Lupus Street), £18 8s. 4d.; Portland Town Dispensary, £21 13s. 4d.; Portobello Road Provident Dispensary, £5 8s. 4d.; Public Dispensary, £59 11s. 8d; Queen Adelaide's Dispensary, £43 6s. 8d.; Royal General Dispensary, £43 6s. 8d.; Royal Maternity Charity, £65; Royal Pimlico Provident Dispensary, £48 15s.; Royal South London Dispensary, £70 8s. 4d.; St. George's and St. James's Dispensary, £59 11s. 8d.; St. George's (Hanover Square) Provident Dispensary, £54 3s. 4d.; St. John's Wood and Portland Town Provident Dispensary, £23 16s. 8d.; St. Marylebone General Dispensary, £37 18s. 4d; St. Pancras and Northern Dispensary, £43 6s. 8d.; SS. Paul and Barnabas Provident Dispensary, £54 3s 4d.; South Lambeth, Stockwell, and North Brixton Dispensary, £59 11s. 8d.; South London Medical Aid Institute, £21 13s. 4d.; Stamford Hill, Stoke Newington, &c., Dispensary, £48 15s.; Tower Hamlets Dispensary, £48 15s.; Wandsworth Common Provident Dispensary, £10 16s 8d.; Westbourne Provident Dispensary and Maternity, £21 13s. 4d., West Dulwich Provident Dispensary, £5 8s. 4d.; Western Dispensary; £43 6s. 8d.; Western General Dispensary, £151 13s. 4d.; West Ham, Stratford, and South Essex Dispensary, £65; Westminster General Dispensary, £43 6s. 8d.

Two hundred and seven pounds in all will be deducted from certain of the above sums, being the amounts received from collections sent direct to particular institutions.

